# Increasing prevalence of hypervirulent ST5 methicillin susceptible *Staphylococcus aureus* subtype poses a serious clinical threat

**DOI:** 10.1080/22221751.2020.1868950

**Published:** 2021-01-17

**Authors:** Ying Jian, Lin Zhao, Na Zhao, Hui-Ying Lv, Yao Liu, Lei He, Qian Liu, Min Li

**Affiliations:** Department of Laboratory Medicine, Renji Hospital, School of Medicine, Shanghai Jiao Tong University, Shanghai, People’s Republic of China

**Keywords:** Methicillin susceptible *Staphylococcus aureus*, phylogenetic analysis, whole-genome sequencing, ST5, virulence

## Abstract

*Staphylococcus aureus* (*S. aureus*) is a clinical pathogen of great significance causing metastatic or complicated infections. ST5 clonotype isolates have dominated *S. aureus* infections for more than 10 years in Shanghai, China, and the proportion of methicillin-susceptible *S. aureus* (MSSA) has remarkably increased in the past decades. By whole-genome sequencing (WGS) 121 ST5 clonotype *S. aureus* isolates using next-generation sequencing (NGS) platforms and characterizing the evolutionary dynamics of ST5 linages, we found that MSSA evolved independently, making it a subtype differed from other MRSA clones. Drug resistance gene analysis by using the NGS data demonstrated that ST5 clonotype MRSA might be more tolerant under the threat of antimicrobials, which was confirmed in further *in vitro* susceptibility tests. However, MSSA subtype isolates exhibited relatively high virulence upon the analysis of virulence factors. Furthermore, MSSA subtype isolates displayed higher hemolysis capacity and higher ability to adhere to epithelial cells including A549 human alveolar epithelial cells and HaCaT human skin keratinocytes, caused more severe infections in murine abscess model. With its high virulence and enhanced magnitude in the past decades, the ST5 MSSA subtype poses a serious clinical threat hence more attention should be paid to the prevention and control.

## Background

*Staphylococcus aureus* (*S. aureus*) is both a biologic pathogen and a symbiotic bacterium. It was one of the first described pathogens, is colonized in about 30% of the healthy non-institutionalized population, and is well adapted to the healthcare environment and its human and animal host [1,2]. It has been considered as a major cause of clinical infections and a severe threat to both communities and healthcare facilities. *S. aureus* can cause metastatic or complicated infections, including pneumonia, bacteremia, osteoarticular infections, endocarditis, and skin and soft tissue infections [3]. According to the latest release by China Antimicrobial Surveillance Network (CHINET, http://www.chinets.com/Data/AntibioticDrugFast), *S. aureus* ranked third in prevalence among all isolated pathogens, accounting for about 9.3% of clinical infections in China in 2019.

A clinical hotspot associated with *S. aureus* is the acquisition of resistance against multiple antibiotics; one of the most commonly described cases is methicillin-resistant *S. aureus* (MRSA). The semisynthetic antibiotic methicillin was introduced for clinical treatment of *S. aureus* infections in the late 1950s, however, a methicillin resistant isolate was after shortly reported by Jevons in England in 1961 [4]. Then MRSA spread globally, causing extensive concern for its serious problems in both the hospital and community settings. The prevalence of MRSA is highly geographically and temporally variable. It ranges from less than 5% in Northern Europe to more than 50% in some American and Asian countries [5–7]. It was once that MRSA counted for 51.6% ICU and 42% non-ICU *S. aureus* infections from early 1998 to mid-2003 in the United States, in comparison to a decline in MRSA prevalence in the past decade [8,9]. Likewise, the percentage of MRSA isolates from hospitalized patients was up to 69.0% when the CHINET project launched in China in 2005. Fortunately, its prevalence has also declined in recent years, and has notably fallen to 31.4% in 2019 in China [10]. *S. aureus* investigations have, however, often underestimated the importance of non-MRSA clones for years, even though the common linage of MSSA is in the process of re-dominating *S. aureus* infections clinically. A portion of studies have turned their attention to specific methicillin-susceptible *S. aureus* (MSSA) linages in recent years [11–13], nevertheless, MSSA infections must be paid more attention.

The pathogenesis of *S. aureus* infections hinges on the production of surface proteins that mediate bacterial adherence to host cells and tissues, as well as the secretion of certain extracellular toxins. Hemolysin, enterotoxin, leukotoxin and toxic-shock syndrome toxin-1 (TSST-1) are common toxins that can induce severe symptoms of infection [14]. Additionally, *S. aureus* manufactures enzymes that destroy host cells and tissues and incapacitate host immune defense, accelerating the growth and spread of bacteria in host cells. Immune evasion cluster (IEC) genes, such as *sak*, *chp*, *scn* and *sea*, are of note conducive for isolates’ escaping from human host’s immune response [15]. The secretion of enzymes such as coagulase, proteases, and staphylokinase enhances the bacteria’s evasion of host defenses, as well as host tissue invasion and penetration. In addition, *S. aureus* surface proteins including clumping factors, fibronectin proteins, protein A and collagen adhesin also facilitate adhesion, tissue invasion, and further host defense evasion [16,17]. Equal to antibiotic resistance ability, the virulence of isolates is of great importance when evaluating an *S. aureus* linage.

Numerous typing techniques can be used to discriminate *S. aureus*. Among these typing methods, Multi-locus Sequence Typing (MLST) has been employed remarkably widely on account of its ease and portability of data comparison in different laboratories [9,18]. The molecular characteristics of MLST make clearer the epidemiology of *S. aureus* infections. Our recent work showed that ST5 type clones dominated in all clinical isolated *S. aureus* in Shanghai in the last decade, indicating that *S. aureus* ST5 merits further attention [19,20]. Furthermore, we found that the percentage of ST5 MSSA subtypes increased in the past decade.

In the research presented herein, we sequenced 123 ST5 clonotype isolates between 2008 and 2018 utilizing the NGS platform, among which the sequence data of 121 isolates, including 79 MRSA isolates and 42 MSSA isolates, passed the quality control procedure. Phylogenetic analysis was then performed to reconstruct the evolutionary dynamics of ST5 clonotype *S. aureus*. The genetic and phenotypic drug resistance and virulence characteristics of these isolates were analyzed, as well. By reconstructing the phylogenetic characteristics and profiling the antibiotics susceptibility and virulence ability, we sought to comprehensively understand the dominating *S. aureus* ST5 linage and determine the trajectory of newly emerging ST5 MSSA subtypes, providing epidemiological evidence for the clinical prevention and control of *S. aureus* infections.

## Materials and methods

### Ethics approval

This study was approved by the Ethics Committee of Renji Hospital, School of Medicine, Shanghai Jiaotong University, Shanghai, China. All individual patients or their legal guardians provided informed consent. This project is a retrospective study. The *S. aureus* isolates from patient samples were cultured and identified in routine microbiology laboratories. All animal experiments were performed following the Guide for the Care and Use of Laboratory Animals of the Chinese Association for Laboratory Animal Sciences (CALAS) and approved by the ethics committee of Renji Hospital, School of Medicine, Shanghai Jiao Tong University, Shanghai, China.

### Bacterial isolates, growth conditions, and clinical definitions

Consecutive and nonrepetitive clinical *S. aureus* isolates were all collected and stored in this affiliated tertiary hospital (a centrally located large and particularly representative teaching hospital with 2000 beds and 10,000 admissions/day) from 2008 to 2018 in Shanghai, China as described before [19]. Randomly selected ST5 clonotype MRSA from year 2008, 2012, 2015 and 2018 by using random selection module in Microsoft Office Excel and all stored ST5 clonotype MSSA isolates from 2008 to 2018 were retrieved (part of the isolates in 2010 and 2012 failed to revive) and grown on sheep blood agar plates or in tryptic soy broth (TSB) (Oxoid). Clinical information was gathered including age, gender of the patients, and specimens from which these clones were isolated.

### Molecular characterization of *S. aureus* isolates

MLST typing of *S. aureus* isolates was carried out by detecting seven housekeeping genes, including *arcC*, *aroE*, *glpF*, *gmk*, *pta*, *tpi*, and *yqiL*, then the sequences of these seven genes were submitted to the *S. aureus* MLST database for the confirmation of ST type(http://www.mlst.net) [21]. Spa typing of the isolates was performed by sequencing the *spa* gene in the polymorphic X region, followed by configuring the data in *S. aureus spa* database to unfold the type (http://www.spaserver.ridom.de) [22].

### Genomic library preparation and whole-genome sequencing, and quality control

Chromosomal DNA of 123 *S. aureus* ST5 isolates was extracted by a standard phenol–chloroform extraction procedure and was used for whole-genome sequencing. Whole-genome sequencing was carried out using the NovaSeq 6000 sequencing platform (Illumina Inc., San Diego, CA) with a 2 × 150 bp read length (BIOZERON Biotechnology, Shanghai, China). Read quality control was conducted with quality control option in CLC Genomics Workbench 12.0(QIAGEN, Aarhus, Denmark) and the raw data were filtered before assembly. In addition, sequences containing more than 10% ambiguous N bases or sequences shorter than 30 bp in length were also removed. Sequencing data of 121 isolates (including 79 MRSA isolates and 42 MSSA isolates) passed the quality control procedure. The Illumina sequences of the remaining 121 isolates in this study are available in the Sequence Read Archive (BioProject accession number: PRJNA660290).

### Trimming, mapping, SNP calling and phylogenetic construction

Trimming of the raw data, whole genome alignment, SNP calling, and phylogenetic analysis were all carried out in CLC Genomics Workbench 12.0 (QIAGEN, Aarhus, Denmark) with the default options. Clean reads were obtained after removing the adapter sequences and low-quality sequences. The whole-genome sequence of the *S. aureus* N315 strain (ST5, GenBank accession code: BA000018.3) was used as the reference template for read mapping. A maximum likelihood (ML) tree using the general time reversible (GTR) model of nucleotide substitution with among-site rate heterogeneity across 4 categories (GTR + Γ) was constructed in CLC Genomics Workbench 12.0 (QIAGEN), followed by annotating using iTOL (https://itol.embl.de/) [23]. The *S. aureus* MW2 strain (ST5, GenBank accession code: BA000033.2) was used as a root when constructing the ML tree.

### De novo assembly, detecting the presence of drug resistance genes and virulence-associated genes

De novo assembly of the clean data was performed in CLC Genomics Workbench 12.0 (Qiagen) using the default options. Then the generated de novo assembled contigs were employed to BLAST at the drug resistant gene database (Resfinder database, 28 May 2020) [24] and at the Virulence Factors Database (VFDB, http://www.mgc.ac.cn/VFs/, May 2019) [25], in order to confirm if the resistant genes or virulence factors were represented there. As IEC genes (such as *sea*, *chp*, *scn* and *sak*) were detected when BLAST at the VFDB, the IEC typing of each isolate was determined as described before [26,27]. *hlb* gene is usually inactivated by the insertion of beta-hemolysin-converting bacteriophages [26]. As the reference *hlb* gene in VFDB only contained sequence encoding beta-hemolysin, BLAST was carried out again using reference *hlb* gene in Genbank (GenBank accession number X13404.1) [28] which contained intact sequence including *hlb* ORF (Open Reading Frame)1, *hlb* ORF2 and beta hemolysin coding sequence against assembled contigs. A tree with drug resistance genes and virulence factors was managed and annotated using iTOL(https://itol.embl.de/) [23].

### In silico sequence type (ST), spa-type, SCCmec type, and prophage identification

WGS data were used for genotypic characterization including the determination of the sequence type (ST) with online tools (https://cge.cbs.dtu.dk/services) including MLST Finder 2.0 for MLST typing [29], *spa*Typer 1.0 for spa typing [30], and SCC*mec*Finder 1.2 (default threshold: 90% identity, 60% minimum length) for SCC*mec* typing. Moreover, SCC*mec* subtyping was carried out using two different methods as described before by International Working Group on the Classification of Staphylococcal Cassette Chromosome Elements and by *Monecke, S* et al. [31,32]. The NGS de-novo assembled contigs were also employed for identifying prophages in PHASTER(http://phaster.ca/) [33,34].

### Antimicrobials resistance profiles

The antimicrobials susceptibility of all isolates was determined by disc diffusion method on Mueller-Hinton agar according to Clinical and Laboratory Standards Institute (CLSI) guidelines [35]. The antimicrobial agents tested included oxacillin, cefoxitin, penicillin, erythromycin, tetracycline, levofloxacin, ciprofloxacin, gentamicin, clindamycin and trimethoprim/sulfamethoxazole (Oxide). *S. aureus* ATCC29213 was used as a quality control.

### Growth curve

Growth curve of *S. aureus* strains (five randomly selected isolates each in the three MRSA clades and eight isolates in the MSSA clades) were carried out as described before [36]. Generally, isolates were grown overnight in 5 ml of TSB with shaking (200 rpm) at 37°C. Overnight cultures were diluted 1:500 in 5 ml of fresh TSB with shaking (200 rpm) at 37°C for a full 24 h. 200 ul of the solution in 1–10 and 24 h was added into a 96-well plate in triplicate and was read with a Micro Enzyme Linked Immunosorbent Assay (Micro-ELISA) Autoreader (Synergy 2) at 600 nm. The solution was diluted 1:10 with fresh TSB before reading at Micro-ELISA when OD_600_ > 1.0.

### Semiquantitative biofilm assay

Semiquantitative biofilm assays were performed as described before [37]. Cells were fixed by Bouin’s fixative. The fixative was removed after incubation of 1 h, then phosphate-buffered saline (PBS) was used to wash each well. Organisms in the wells were later stained with crystal violet, and the floating stain was washed off with slow-running water. After drying, the stained biofilm was read with a Micro-ELISA Autoreader (Synergy 2) at 570 nm. The assay was performed in triplicate.

### Lysis of erythrocytes by culture filtrates

Erythrocytes lysis tests were carried out as described before [38]. *S. aureus* isolates were grown on TSB cultures for 15 h, then the supernatants were collected for later use. Hemolytic activities were identified by incubating supernatant samples with 2% human red blood cells in PBS for 1 h at 37°C. Ultimately, hemolysis was determined by measuring the optical density at 540 nm using Micro ELISA Autoreader (Synergy 2). The assay was performed in triplicate.

### Adhesion of *S. aureus* to human alveolar A549 and human skin epithelial cells HaCaT

Cell adhesion assay was conducted as described before [39]. Human alveolar epithelial cells A549 and skin epithelial cells HaCaT were culture in Dulbecco’s Modified Eagle Medium (DMEM/High Glucose) with fetal bovine serum (FBS, 10%) and penicillin–streptomycin solution (0.5%), at 37 °C and 5% CO2. ST5 clonotype *S. aureus* isolates (five randomly selected isolates each in the three MRSA clades and eight isolates in MSSA clade) were grown to the midlogarithmic growth phase, followed by two-time wash with DMEM medium. Cells and bacteria were mixed at a ratio of 1:10 (multiplicity of infection, MOI = 10), and then incubated for 2 h. For the sake of removing non-adherent bacteria, supernatants were discarded and cells were washed three times with sterile PBS. Cells were subsequently lysed using 0.1% deoxy-sodium cholate solution. Ultimately bacterial CFU were enumerated by serial dilutions of epithelial cell lysates and plating onto sheep blood agar plates.

### Mouse skin abscess model

Mouse skin abscess assays were performed as described before [38]. Outbred, immunocompetent hairless female mice between 4 and 6 weeks of age were used for the abscess model. Overnight cultures (grown in TSB) of the randomly selected 4 isolates each group in the three MRSA clades in MSSA clade were sub-cultured (1:100) in fresh TSB for and grown for 4 h. After centrifugation at 5000 × g for 10 min, cells were washed twice and resuspended in PBS. Mice were anesthetized with 2,2,2-tribromoethanol (Sigma-Aldrich) and subcutaneously inoculated with 50 μL PBS containing 10^7^ live *S. aureus* in the back skin. The abscess length (L) and width (W) dimensions were used to calculate the abscess area using the formula π × (L × W) after 24 h.

### Statistical analysis

Unpaired two-tailed Student’s *t*-tests and chi-square test or Fisher exact test were performed to analyze statistical significance. All data were analyzed using GraphPad Prism 8.0. Error bars in all graphs indicated the standard deviation (mean ± SD), and *P* values <0.05 were reported as statistically significant.

## Results

### Percentage of ST5 MSSA subtypes increased in 2008–2018

**Figure 1.**
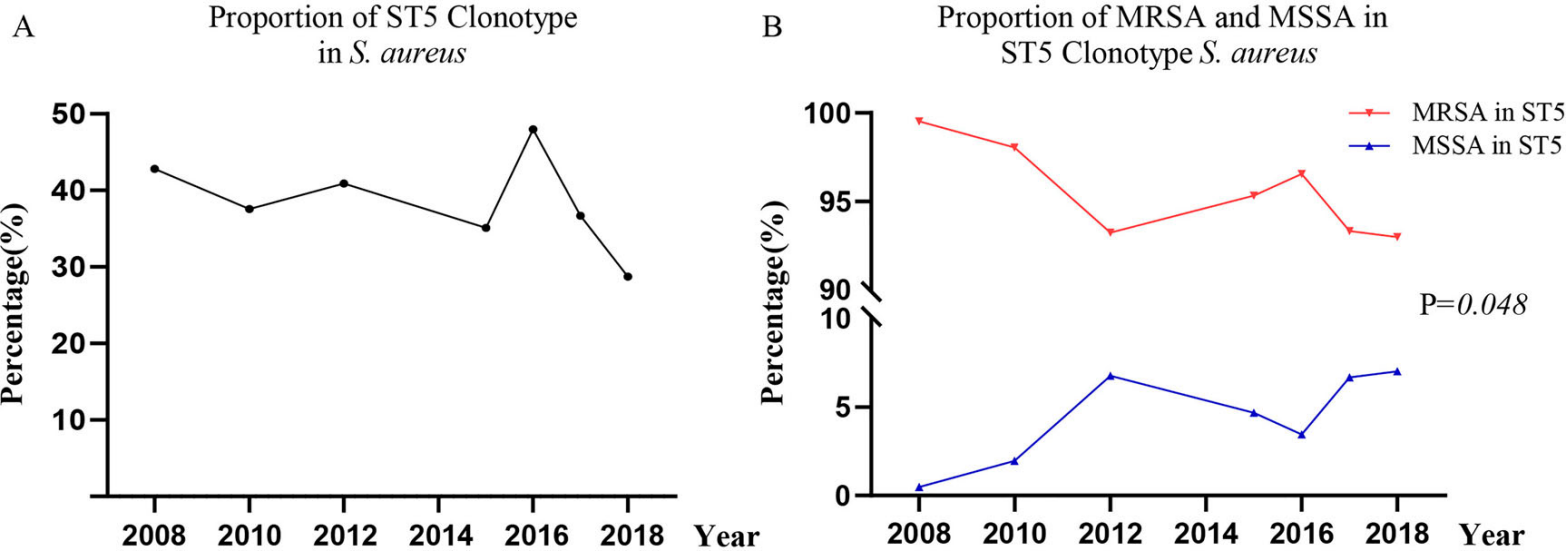
Dynamic changes in proportion of ST5 clonotype in *S. aureus* (A) and in proportion of MRSA and MSSA in ST5 clonotype *S. aureus* (B).

In our prevalence characterization survey of *S. aureus* in Shanghai, China, we found that from 2008 to 2018 the percentage of ST5 clonotype isolates in all *S. aureus* was maintained at a relatively high level, basically with a proportion of more than 35% every year (Figure 1A). The percentage of MRSA was reduced in the past decade (Figure 1B, from 99.51% in 2008–92.98% in 2018, *P* = *0.048*), and correspondingly the percentage of MSSA was augmented (Figure 1B, from 0.49% in 2008 to 7.02% in 2018, *P* = *0.048*).

### Demographic characteristics of the selected ST5 clonotype isolates

In this phylogenetic analysis, 123 isolates (randomly selected MRSA isolates and all stored MSSA isolates) were chosen to investigate the evolutionary dynamics of ST5 clonotype *S. aureus*. The NGS data of 2 isolates were out of quality control, hence data of the remaining 121 isolates (79 MRSA isolates and 42 MSSA isolates) were gathered to construct the phylogenetic characteristics. Relative information of these 121 clinical *S. aureus* isolates is listed in Table 1. When stratified by gender, male patients were found to be more susceptible to *S. aureus* infections than female (65.82% versus 34.18% in MRSA and 71.43% versus 28.57% in MSSA). Patients with *S. aureus* infections were more likely to be older than 20(97.47% in MRSA and 97.62 in MSSA), of which senior patients played a crucial part (age ≥ 65, 35.44% in MRSA and 42.86 in MSSA). No significant difference was found between MRSA and MSSA in gender constitution and in age composition. *S. aureus* specimens isolated were mainly from the respiratory system (82.28% in MRSA and 42.86% in MSSA, *P* < *0.001*). Additionally, skin and soft tissue infections accounted for about 28.57% of all MSSA infections and 7.59% of all MRSA infections (*P* = *0.002*). Other specimens such as catheter, blood, urine, and other sterile body fluids comprised a limited amount of the selected 121 samples.

**Table 1.** Constitution in gender, age group, and specimen sources of the 121 ST5 clonotype *S. aureus* isolates.

Item	Group	Number (Percentage (%))		
		MRSA	MSSA	*P*
Gender	Male	52(65.82%)	30(71.43%)	*0.530*
	Female	27(34.18%)	12(28.57%)	
Age group	≤20	2(2.53%)	1(2.38%)	*0*.*746*
	21–64	49(62.03%)	23(54.76%)	
	≥65	28(35.44%)	18(42.86%)	
Specimen sources	Respiratory system	65(82.28%)	18(42.86%)	**<*0***.***001***
	Skin/soft tissue	6(7.59%)	12(28.57%)	***0***.***002***
	Catheter	3(3.80%)	0(0%)	*0*.*551*
	Blood	1(1.27%)	3(7.14%)	*0*.*120*
	Other sterile body fluids	2(2.53%)	2(4.76%)	*0*.*609*
	Urine	2(2.53%)	2(4.76%)	*0*.*609*
	Others	0(0%)	5(11.90%)	*0*.*004*
No. of total	Total	79	42	

### Phylogenetic construction of ST5 clonotype *S. aureus*

**Figure 2.**
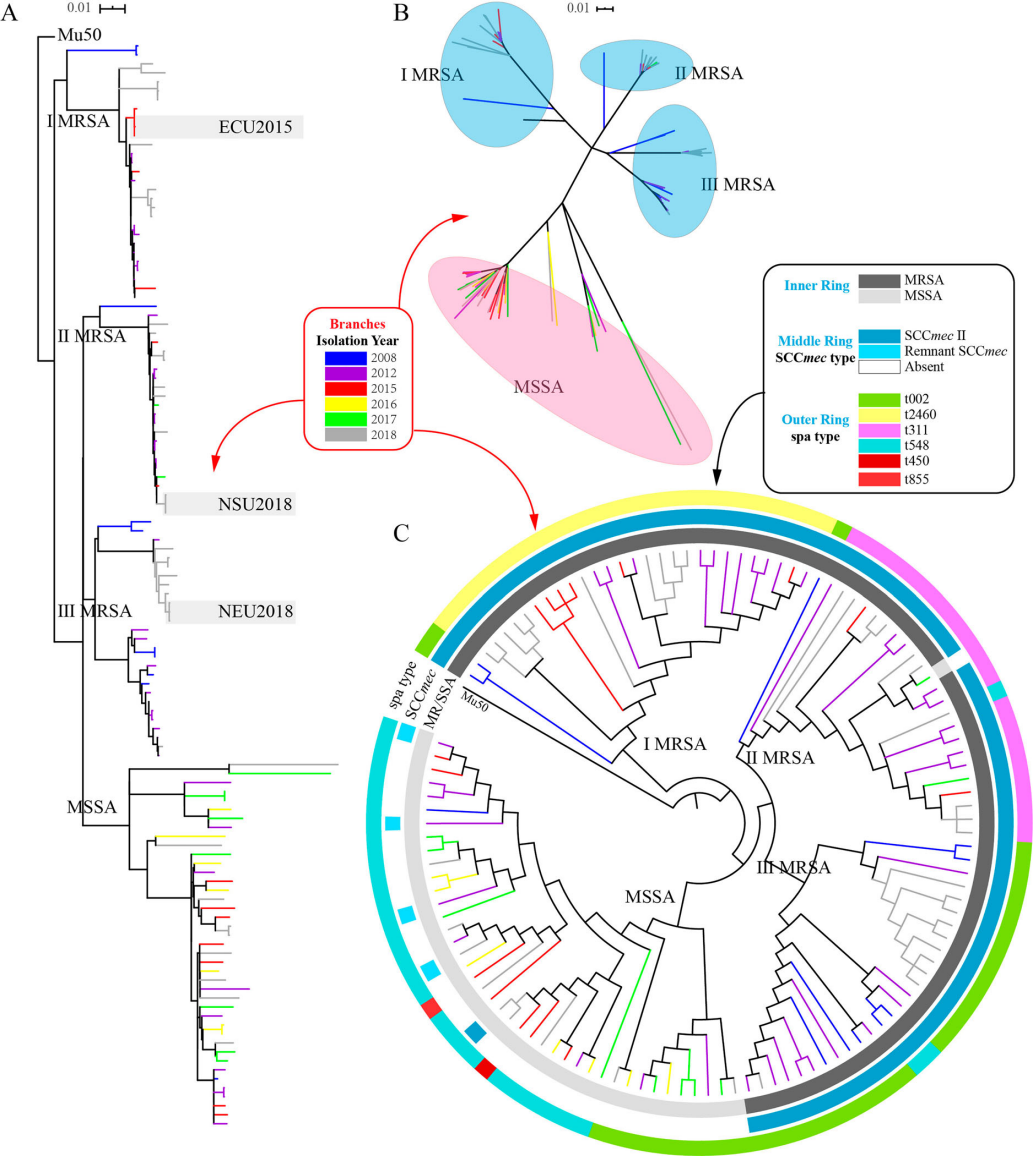
Phylogenetic structure of ST5 clonotype *S. aureus* isolates. Years are differentiated by branches’ colours in the phylogenetic tree. (**A**) Maximum likelihood tree of ST5 clonotype *S. aureus* isolates. This tree was built using a maximum likelihood method with GTR substitution model utilizing SNPs of the 121 isolates and Mu50 *S. aureus* isolate, the NGS raw data of which were mapped with genome of *S. aureus* reference N315. The tree is rooted with Mu50 as an out group. Three groups of independent epidemics (paired SNPs of isolates ≤ 4 were defined as clones in an independent epidemic). (**B**) Unrooted tree of ST clonotype *S. aureus* isolates. Branches in this tree could be classified into 4 clades, including Clade I MRSA, Clade II MRSA, Clade III MRSA and Clade MSSA. (**C**) Classification of MRSA/MSSA (inner ring), SCC*mec* type (middle ring), spa type (outer ring) of the 121 isolates. Colours to distinguish each type are shown above.

6466 core genome SNPs between the 121 ST5 clonotype isolates were identified and used to construct a maximum likelihood tree (Figure 2A). The population framework revealed a diverse population structure containing several distinct clades, including three MRSA clades and one MSSA clade. The four clades could be markedly distinguished in the unrooted tree (Figure 2B). The three MRSA clades were named Clade I MRSA, Clade II MRSA, and Clade III MRSA, while the MSSA clade was named Clade MSSA. Mapping of the isolates revealed the independent evolution of MRSA and MSSA within each clade in ST5 clonotype *S. aureus*, proving that the ST5 MSSA linage was a new subtype that differed from other ST5 MRSA isolates. Five ST5 isolates which carried the *mecA* gene were susceptible to oxacillin and cefoxitin, and we classified them into ST5 Clade MSSA group on the basis of phylogenetic analysis. Moreover, as the colours of the branches show, there was no evidence that proved that neighbouring clades or branches had association with their isolation years.

Paired distance between isolates was of importance to define whether the isolates were distantly or closely related, so we defined the paired distance between all isolates in the project and the result was showed in Figure S1. Generally, paired distance of isolates in MRSA Clade (including Clade I MRSA, Clade II MRSA and Clade III MRSA) was from 0 to 588 (with a median of 408 and an average of 352), while the number was between 0 and 823 (with a median of 203 and an average of 322) in ST5 MSSA isolates. Of note was that the paired distance between five oxacillin-susceptible *mecA*-positive isolates was from 63 to 193 (with a median of 147.5 and an average of 152). Isolates with a paired distance less than or equal to 4 SNPs were deemed to be from the same transmission [40]. Three independent transmissions of small scale were identified in this analysis and are shown in Figure 2A: In ECU 2015 were clones isolated from three patients in the Emergency Care Unit (ECU) in 2015; three isolates in NSU2018 were collected in three patient from the Neurosurgery Unit in 2018; three branches in the near position of group NEU2018 were two clones isolated in the Neurology Unit and one isolate from the Internal Medicine Department in 2018.

Characteristics of isolates’ susceptibility or resistance to methicillin are shown in the inner ring in the circular tree (Figure 2C). Similarly, SCC*mec* type of these isolates are shown in the middle ring (Figure 2C). All ST5 clonotype MRSA in this analysis shared the same type: SCC*mec*II. Interestingly, it revealed that all MRSA isolates were subtyped as SCC*mec* II (N315) using two different methods described before [31,32]. We analyzed these isolates and found that four of them carried remnant SCC*mec* (information was listed in Supplementary Table 1, as well). What interested us was that an isolate with intact SCC*mec* II exhibited high susceptibility to oxacillin and cefoxitin, even if the induced oxacillin resistance test was performed. The five strains were classified into Clade MSSA as shown in the maximum likelihood tree. Constitution of *spa* type of the 121 isolates is shown in the outer ring (Figure 2C): t002 (38 isolates, 31.4%), t2460 (27 isolates, 22.3%), t311 (22 isolates, 18.2%), t548 (32 isolates, 26.4%), and only one t450 MSSA strain and one t855 MSSA isolate.

### MSSA exhibited different drug resistance profiles in comparison to MRSA

**Figure 3.**
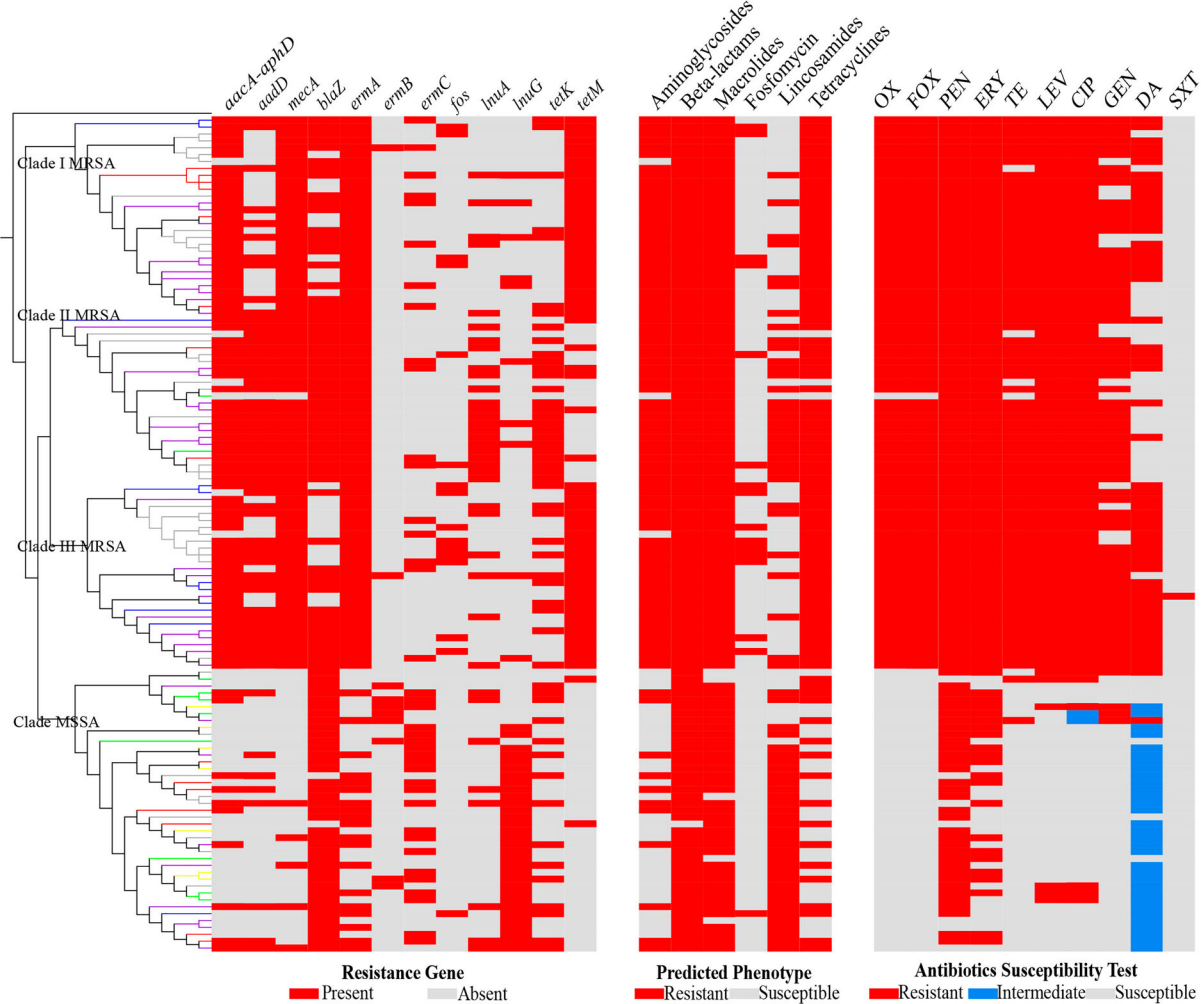
Resistance gene (left), predicted phenotype (middle) (all conducted in CLC Genomics Workbench 12.0 using ResFinder database), and antibiotics susceptibility profiles (right) of the 121 ST5 clonotype *S. aureus* isolates (from top to bottom are isolates in Clade I MRSA, Clade II MRSA, Clade III MRSA and Clade MSSA, respectively).

Numerous drug resistance genes were detected using NGS data. The presence or absence of drug resistance gene (left), predicted phenotype (middle) and antibiotics profile (right) of each isolate is shown in Figure 3. Aminoglycoside resistance genes including *aacA-aphD* and *aadD* were detected in more MRSA isolates than MSSA (93.67% versus 23.81%, *P* < *0.001*; 67.09% versus 19.05%, *P* < *0.001*); *mecA* presented in all MRSA isolates and five MSSA isolates which were susceptible to cefoxitin and oxacillin, even though oxacillin induction test was carried out; *blaZ* which encodes resistance to beta-lactams, presented in most ST5 isolates regardless of the methicillin susceptibility, and displayed an even lower proportion in MRSA (73.42% in MRSA versus 95.24% in MSSA, *P* = *0.004*). Macrolides resistance genes *ermA*, *ermB* and *ermC* exhibited differing profiles in MRSA and MSSA (100% versus 47.62%, *P* < *0.001*; 2.53% versus 19.05%, *P* = *0.003*; and 21.52% versus 52.38%, *P* = *0.001*, respectively). Lincosamides resistance gene *lnuA* was detected in more MRSA (36.71%, especially in Clade II MRSA72.73%) versus MSSA(19.05%; MRSA versus MSSA, *P* = *0.045*; Clade II MRSA versus Clade MSSA, *P* < *0.001*),while *lnuG* was detected in more MSSA than MRSA (76.19% versus 12.66%, *P* < *0.001*); *tetK* was found to be present in more Clade II MRSA isolates (77.27% versus 24.14% in Clade I MRSA, 44.83% in Clade III MRSA and 36.59% in Clade MSSA; Clade II MRSA versus Clade MSSA, *P* = *0.002*), while *tetM* was detected in most Clade I and III MRSA (100%, 93.10% versus 27.27% in Clade II MRSA, 4.88% in Clade MSSA; MRSA versus MSSA *P* < *0.001*).

The predicted resistance proportion of ST5 clonotype isolates to some antibiotics varied between MRSA and MSSA as well. Generally speaking, more MRSA isolates were predicted to be resistant to aminoglycosides and tetracyclines compared with MSSA (97.47% versus 26.19%, *P* < *0.001*; and 97.47% versus 40.48%, *P* < *0.001*), while this discrepancy was reversed for lincosamides (82.93% with MSSA versus 41.29% with MRSA, *P* < *0.001*; of note was the proportion in Clade II MRSA, which reached up to 77.27%). Nearly all ST5 clonotype isolates exhibited predicted resistance to beta-lactams (100% in MRSA and 97.62% in MSSA, *P* = *0.347*) and macrolides (100% in MRSA and 88.10% in MSSA, *P* = *0.004*), whereas they were predicted susceptible to fosfomycin (2.38% in MSSA versus 16.46% in MRSA, *P* = *0.033*).

Next, we profiled the 121 ST5 clonotype *S. aureus* isolates with *in vitro* susceptibility tests to ten common antibiotics. Oxacillin and cefoxitin, two antibiotics used to define MRSA and MSSA clinically, exhibited identical characteristic in these ST5 clonotype isolates: MRSA isolates were all resistant to oxacillin and cefoxitin, while MSSA isolates were all susceptible. The non-susceptible rate of the ten antibiotics in MRSA and MSSA were as follows: oxacillin(100% versus 0, *P* < *0.001*), cefoxitin (100% versus 0, *P* < *0.001*), penicillin(100% versus 83.33%, *P* < *0.001*), erythromycin(100% versus 54.76%, *P* < *0.001*), tetracycline(96.20% versus 4.76%, *P* < *0.001*), levofloxacin(100% versus 16.67%, *P* < *0.001*), ciprofloxacin(100% versus 21.43%, *P* < *0.001*), gentamycin(84.81% versus 9.52%, *P* < *0.001*), clindamycin(68.35% versus 80.95%, *P* = *0.139*), trimethoprim/sulfamethoxazole (1.27% versus 0, *P* = *1.000*).

### MSSA isolates exhibited higher virulence gene carriage in comparison to MRSA

**Figure 4.**
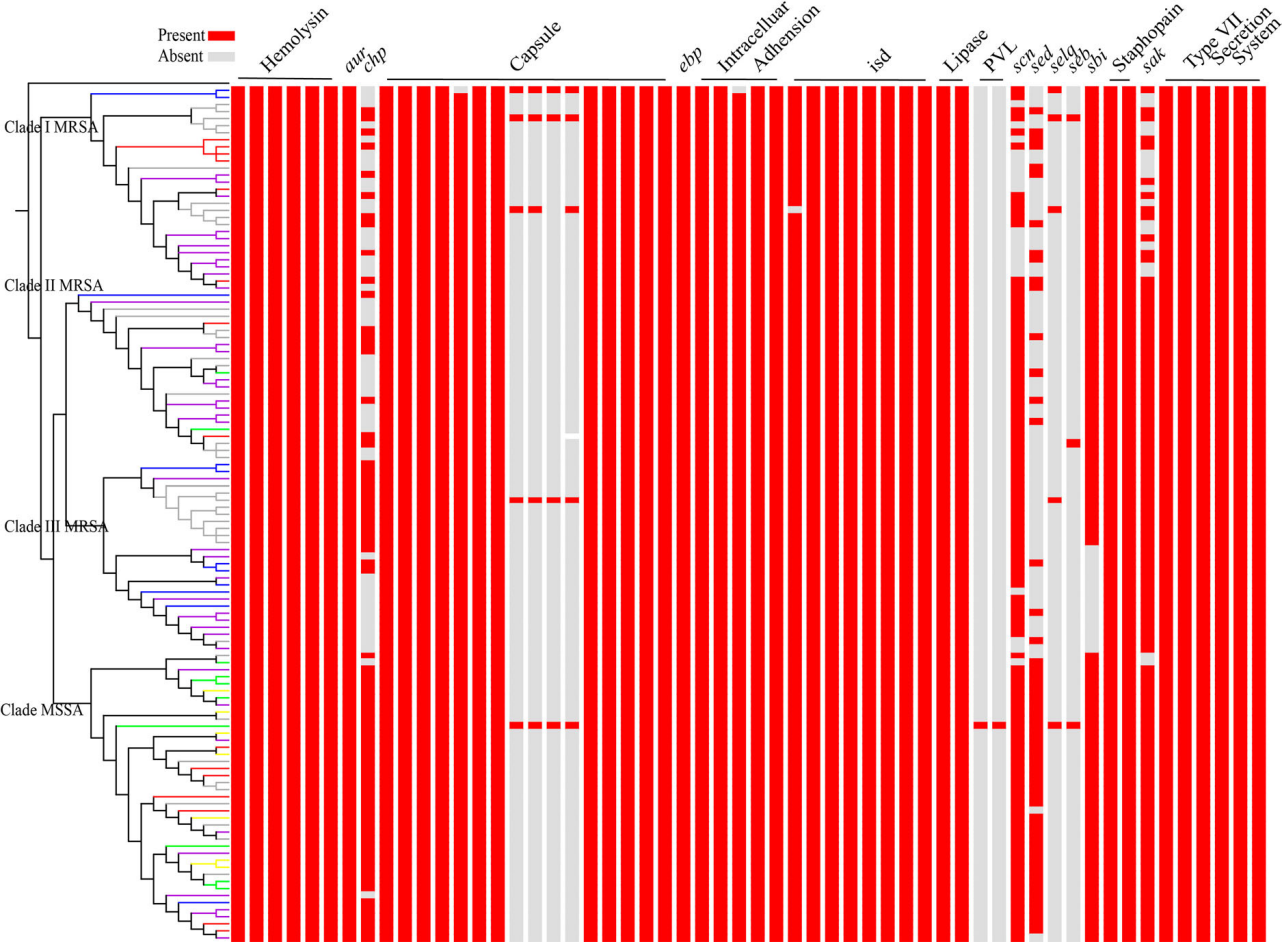
Virulence characteristics of the 121 ST5 clonotype isolates (conducted in CLC Genomics Workbench 12.0 using VFDB database). Absence or presence of the virulence factors in the four clades of *S. aureus* isolates (from top to bottom are isolates in Clade I MRSA, Clade II MRSA, Clade III MRSA and Clade MSSA, respectively).

A series of virulence genes were analyzed using NGS data as shown in Figure 4. Hemolysin associated *hla, hlb, hld, hlgA, hlgB, hlgC* were found to be present in all isolates, Notably, all isolates harboured truncated *hlb* gene as intact *hlb* ORF1 was not detected in each isolate. Capsule associated *cap8A, cap8B, cap8C, cap8D, cap8E, cap8F, cap8G, cap8P, cap8L, cap8N, cap8O* existed in all isolates, while *capH, capI, capJ, capK* were absent from most ST5 isolates. The *aur* gene, which encodes aurolysin in *S. aureus*, presented in all isolates as well. Virulence factors related to intracellular adhesion system, iron-regulated surface determinants (isd) and type VII secretion system associated genes showed no significant difference between MRSA and MSSA as nearly all isolates carried these. Of note were *chp, scn, sed* and *sak,* which displayed statistically difference between MRSA and MSSA in ST5 clonotype isolates (respectively, 41.77% versus 92.86%, *P* < *0.001*; 75.95% versus 97.62%, *P* = *0.002*; 21.52% versus 92.86%, *P* < *0.001*; and 81.01% versus 95.24%, *P* = *0.032*).

IEC genes such as *chp*, *scn* and *sak* were detected in many isolates in this study, so IEC types of all the isolates were distinguished and the result was listed in Supplementary Table 1. Type B constituted the main part of isolates (n = 56, 46.28%), type A, C, D, E were assigned about 6.61%(n = 8), 2.48%(n = 3), 18.18%(n = 22) and 8.26%(n = 10) of the isolates. When we analyzed discrepancy of the constitution between MRSA isolates and MSSA isolates, we found that MSSA isolates were more likely to be IEC type B (83.3%, n = 35), while type B and type D counted 26.58%(n = 21) and 25.32%(n = 20) of all MRSA isolates.

Additionally, we detected prophages of all *S. aureus* ST5 isolates in PHASTER; results are listed in Supplementary Table 1. PHASTER detected 1.736 ± 0.704 intactprophages, 0.6 ± 0.623 questionable prophages and 2.521 ± 1.432 incomplete prophages in each isolate on average. When we analyzed discrepancy of the prophages between MRSA isolates and MSSA isolates, we found that MRSA harboured more prophages than MSSA (5.684 ± 1.410 versus 3.357 ± 1.186, *P* < *0.001*).

### ST5 MSSA subtype isolates displayed hypervirulent in vitro and in vivo

**Figure 5.**
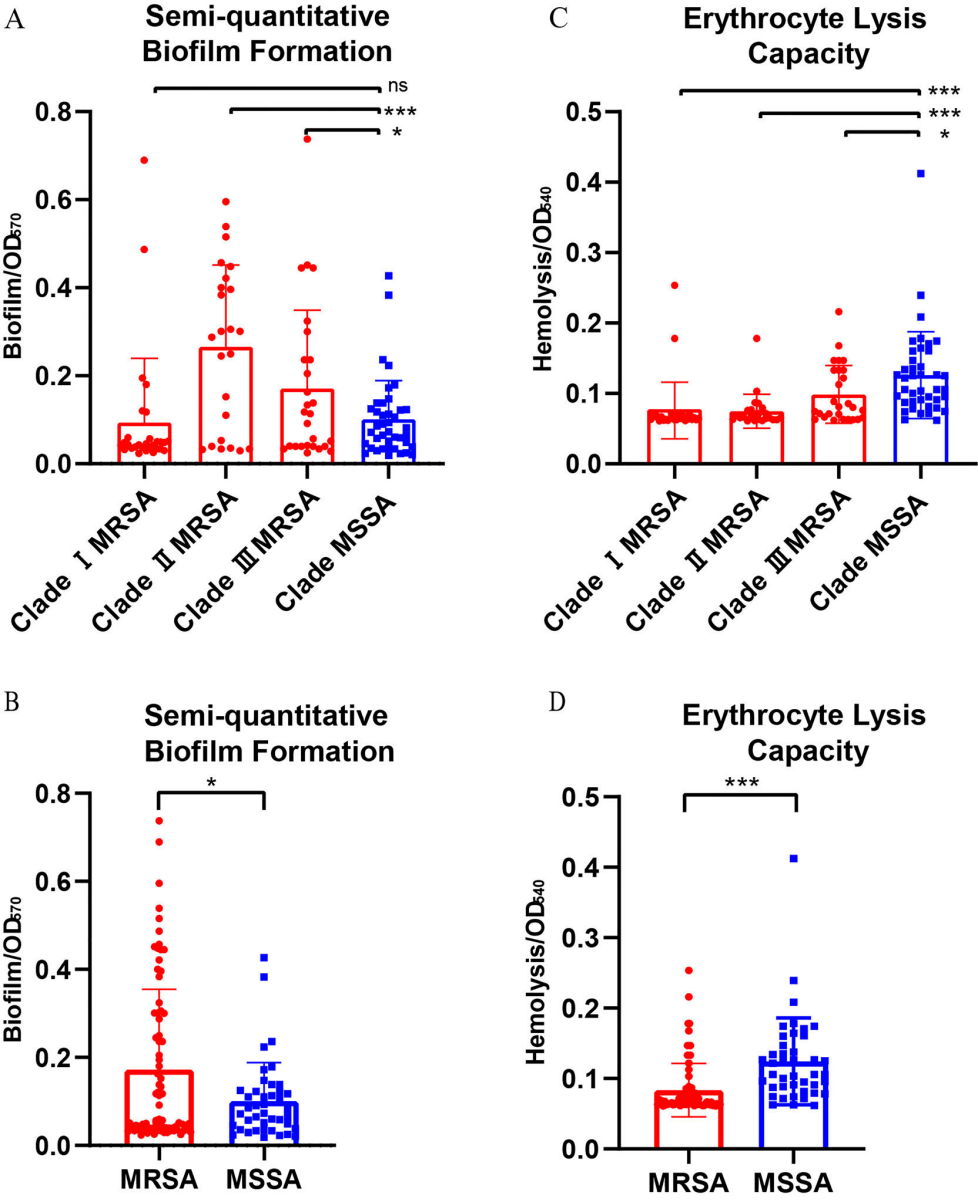
Semiquantitative biofilm formation ability (separated by four clades(**A**) or by MRSA/MSSA(**B**)) and erythrocyte lysis capacity (separated by four clades(**C**) or by MRSA/MSSA(**D**)) of the 121 ST5 clonotype *S. aureus* isolates.

Semi-quantitative biofilm tests and erythrocyte lysis assays were carried out on all the 121 ST5 clonotype isolates, as shown in Figure 5. MSSA showed high erythrocyte lysis capacity in comparison with MRSA (OD_540_: 0.1245 ± 0.0617 versus 0.0836 ± 0.0379, *P* < *0.001*); when the isolates were divided by clades, we found significant difference between Clade I MRSA and Clade MSSA (OD_540_: 0.0760 ± 0.0402 versus 0.1260 ± 0.0617, *P* < *0.001*), and between Clade II MRSA and Clade MSSA (OD_540_: 0.0749 ± 0.0242 versus 0.1260 ± 0.0617, *P* < *0.001*). On the contrary, MRSA exhibited higher biofilm formation ability compared with MSSA (OD_570_: 0.1721 ± 0.1827 versus 0.1010 ± 0.0874, *P* = *0.019*). Of note, Clade II MRSA isolates exhibited statistically higher biofilm formation ability than isolates in Clade I MRSA and Clade MSSA (OD_570:_ 0.2654 ± 0.1864(Clade II MRSA) versus 0.0939 ± 0.1459(Clade I MRSA), *P* < *0.001*; and versus 0.1008 ± 0.0885(Clade MSSA), *P* < *0.001*). Moreover, in order to determine whether MSSA exhibited growth ability that varied in comparison with MRSA, growth curves of randomly selected isolates from the four clades were constructed (Figure S2), while no significant difference was found between ST5 MSSA and MRSA.

**Figure 6.**
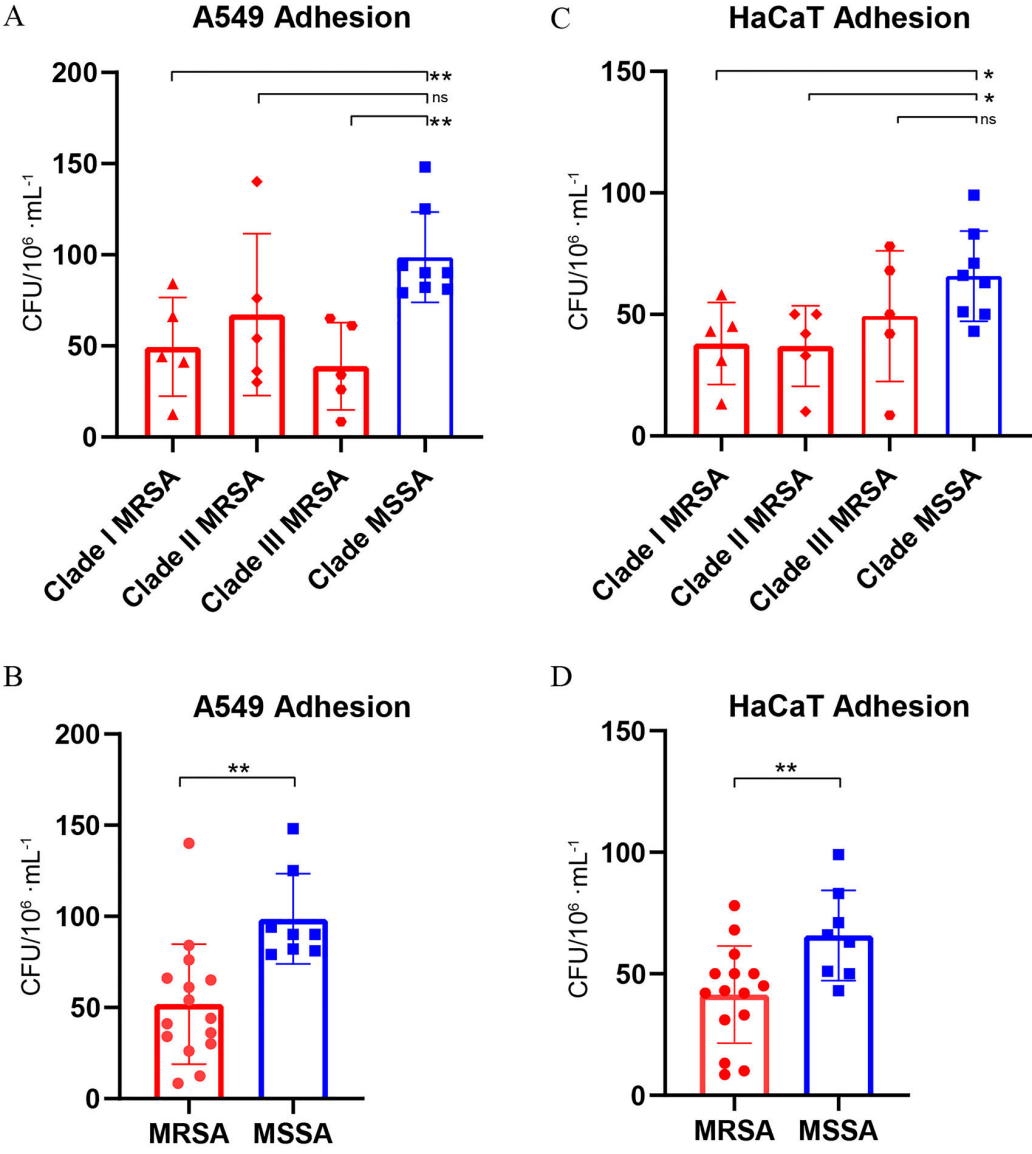
Cell adhesion assays in Human alveolar epithelial A549 cells (separated by four clades(**A**) or by MRSA/MSSA(**B**)) and human skin keratinocytes HaCaT (separated by four clades(**C**) or by MRSA/MSSA(**D**)) of randomly selected ST5 clonotype *S. aureus* isolates (5 randomly selected isolates each in Clade I, II and III MRSA, respectively, and 8 isolates in Clade MSSA).

Respiratory tract infections and skin/soft tissue infections were the most commonly diagnosed *S. aureus* infections clinically. Human alveolar epithelial A549 cells and human skin keratinocytes HaCaT were cultured and cell adhesion assays were performed using randomly selected isolates from four clades. We found that MSSA isolates showed higher adhesion ability than MRSA isolates in both A549 and HaCaT cells (Figure 6B, CFU/10^6^·mL^−1^: 98.63 ± 24.72 versus 51.85 ± 32.94, *P* = *0.002*; and Figure 6D: 98.63 ± 24.72 versus 41.45 ± 19.99, *P* = *0.010*). When stratified by clades, significant difference was found between Clade I MRSA isolates and Clade MSSA isolates (Figure 6A: CFU/10^6^·mL^−1^: 49.48 ± 27.12 versus 98.63 ± 24.72, *P* = *0.006*), and between Clade III MRSA isolates and Clade MSSA isolates (Figure 6A: CFU/10^6^·mL^−1^: 38.88 ± 23.93 versus 98.63 ± 24.72, *P* = *0.001*) in A549 cells. Furthermore, a significant difference was found between Clade I MRSA isolates and Clade MSSA isolates (Figure 6C: CFU/10^6^·mL^−1^: 38.04 ± 16.87 versus 65.75 ± 18.60, *P* = *0.021*), and between Clade II MRSA isolates and Clade MSSA isolates (Figure 6C: CFU/10^6^·mL^−1^: 37.02 ± 16.60 versus 65.75 ± 18.60, *P* = *0.017*), demonstrating that Clade MSSA isolates were more prone to adhere to HaCaT cells than Clade I, II MRSA isolates. Briefly, isolates from ST5 MSSA subtype were of higher cell adhesion capability than ST5 MRSA isolates.

**Figure 7.**
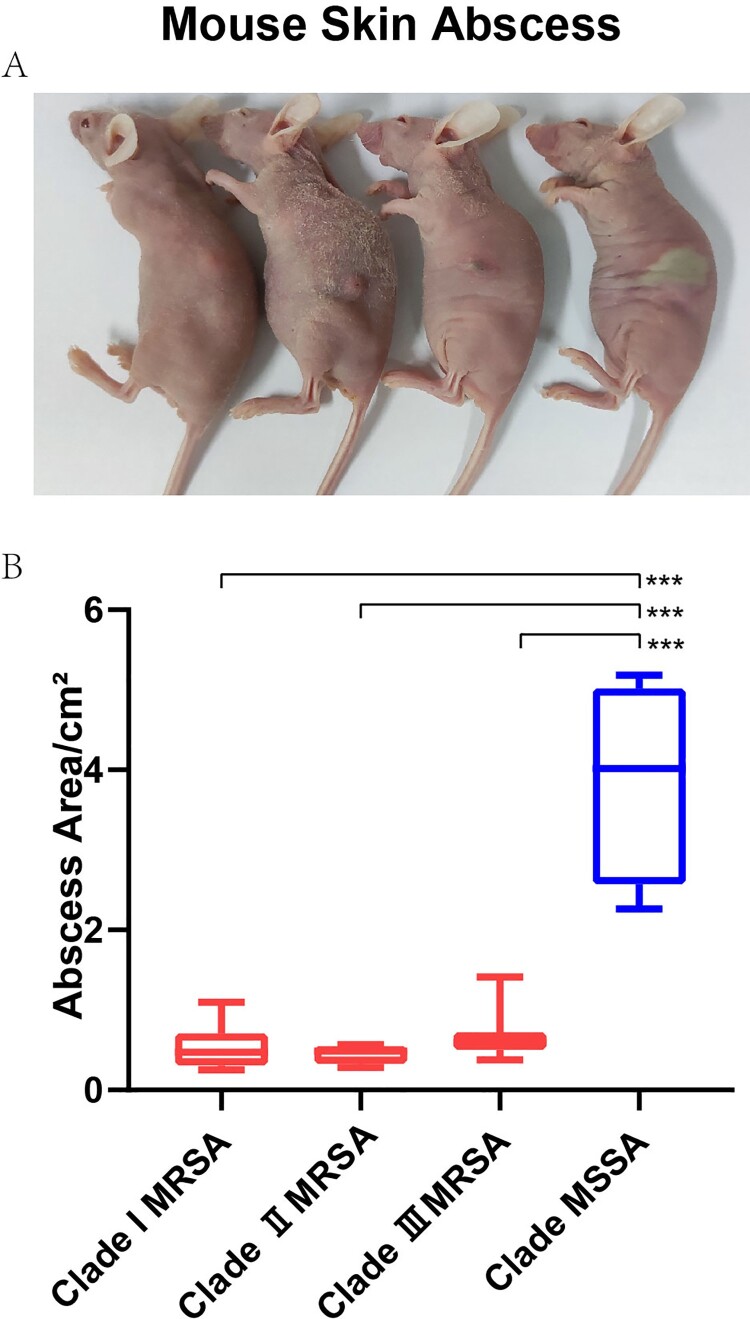
Mouse skin abscess infections caused by isolates from four clades. Randomly selected four isolates in each clade were used for infections in mouse back skin. (**A**)Representative mouse back skin abscess after 24 h inoculation of isolates from 4 Clades. From left to right were mice infected with *S. aureus* isolates in Clade MRSA I, Clade MRSA II, Clade MRSA III, Clade MSSA. (**B**) Back skin abscess areas of 16 mice (4 mice for each group) using the formula π × (L × W) after 24 h’ infection with isolates from four clades.

After evaluating the discrepancy of Clade MRSA and Clade MSSA isolates on the capacity of biofilm formation, erythrocyte lysis, and epithelial cell adhesion *in vitro*, we then explored its virulence potential *in vivo* using mouse skin abscess model. After subcutaneously inoculated with 50 μL PBS containing 10^7^ live *S. aureus* in the back skin for 24 h, the abscess area (π × (L × W)) was showed in Figure 7A and B. MSSA isolates caused a significantly larger abscess area than MRSA isolates (3.87 ± 1.27 in Clade MSSA isolates versus 0.53 ± 0.28 in Clade I MRSA isolates and 0.46 ± 0.12 in Clade II MRSA isolates, and 0.67 ± 0.32 in Clade III MRSA isolates).

## Discussion

Recent epidemiological data have indicated that ST5 dominated *S. aureus* infections in the past decade in Shanghai; furthermore, we found that there was a significant enhancement of the proportion of MSSA isolates in ST5 clonotype *S. aureus* [19,20]. Similarly, a noticeable alteration was that the proportion of MRSA in *S. aureus* infections declined remarkably in accordance with surveillance by CHINET [10], which might be interpreted as a result of the rational and rigorous management of antibiotics in China. Issued in 2012 by the Ministry of Health of the PRC, “Regulations for Clinical Application of Antibacterial Agents” provided a legal guarantee for the rational use of antibacterial agents in China and, as a result of which, clinical medication achieved standardization pledging that antimicrobials resistance could become steerable [41].

Phylogenetic reconstruction of *S. aureus* clonotypes ST8, ST59, ST398, ST188 and some other community associated linages have been well described [38,39,42,43], while the evolutionary dynamics of the dominating pandemics healthcare associated ST5 clonotype *S. aureus* has rarely been studied. We reconstructed the phylogeny of ST5 linages using isolates collected in an affiliated tertiary hospital from 2008 to 2018, with which we were able to shed light on the origin and evolution of ST5 clonotype MSSA in Shanghai, China. Four clades that displayed in the phylogenetic tree that covered 121 individual *S. aureus* isolates distinguished MRSA and MSSA readily, proving that MSSA isolates evolved independently as an emerging ST5 subtype that differed from those dominating MRSA isolates in clinical *S. aureus* infections.

MSSA isolates definitely exhibited differences both genetically and phenotypically in comparison to MRSA isolates. Herein analysis of the resistance genes, prediction of resistance phenotype by using these genes, as well as the phenotype definitely exhibited via *in vitro* antibiotic susceptibility testing all revealed that methicillin susceptible ST5 clonotype isolates exhibited high sensitivity to several common antibiotics in comparison to methicillin resistant ones. First-line antibiotics like beta lactams exhibited various antibacterial ability: MSSA isolates were susceptible to oxacillin while most of them were resistant to penicillin, which might be relevant to the iterative updating of antibiotics. MSSA isolates also showed higher sensitivity to aminoglycosides, tetracyclines and macrolide antibiotics, providing more treatment options even though the number of isolates of ST5 MSSA subtype had risen over the collection period. On the contrary, MRSA isolates were resistant to the majority of first-line antibiotics, but the overwhelming plurality were susceptible to trimethoprim/sulfamethoxazole, a compound of two synthetic antibiotics, providing insight for the instruction of empirical clinical medication for suspicious infections prior to receiving the report of *in vitro* antimicrobials susceptibility tests from a clinical microbiology laboratory.

Notably, human *S. aureus* clones harbour IEC on a prophage that is stably integrated into the *hlb* gene on the bacterial chromosome [44]. IEC elements encodes immune modulators, including staphylococcal enterotoxin A, staphylococcal enterotoxin P, staphylokinase, staphylococcal complement inhibitor, and chemotaxis inhibitory protein of staphylococci, which interact specifically with components of the human innate immune system [15]. IEC genes are usually not found animal staphylococci [45], associated to the high detection rate in our study as the isolates were basically from human host. Staphylokinase encoded by s*ak* is a plasminogen activator secreted by the majority of *S. aureus* isolates [46]. Staphylokinase forms complexes with trace amounts of plasmin present in host plasma, and it subsequently cleaves plasminogen, resulting in the promotion of bacterial entry and further spread in the skin [47,48]. Investigation has suggested that staphylokinase prevents biofilm formation, which might explain the higher biofilm formation ability of MRSA isolates due to their relatively low rate of *sak*’s presence [49]. *scn* is able to encode staphylococcal complement inhibitor (SCIN), a 10 kDa protein found in 90% of clinical *S. aureus* strains that specifically interacts with bacterium that bind C3 convertases and thus efficiently prevents C3b deposition and phagocytosis [50]. The chemotaxis inhibitory protein of staphylococci (CHIPS) encoded by *chp* are produced by 65% of clinical *S. aureus* strains. CHIPS is a 14 kDa protein that blocks neutrophil chemotaxis by binding the formylated peptide receptor and the C5a receptor on neutrophils [51,52]. SCIN and CHIPS are both efficient modulators of neutrophil chemotaxis, phagocytosis and killing, and their early expression is necessary for efficient modulation of the early immune response [53]. *sed*, located on a large penicillinase-type plasmid in *S. aureus* which differs from the bacteriophage-located *sea*, encodes staphylococcal enterotoxin type D, which is associated with food poisoning. Higher carriage rate of *chp*, *sak*, and *scn* in these isolates might indicate a higher ability of immune escape of ST5 MSSA during host defense in *S. aureus* infections.

In addition to the high presence of virulence genes, MSSA isolates exhibited higher hemolysis capacity and higher adhesion ability to the epithelial cells A549 and HaCaT. In the meanwhile, *in vivo* murine abscess model revealed that ST5 MSSA isolates could cause more severe skin infections in comparison to MRSA isolates. All these indicated that ST5 MSSA subtype was determined as a hypervirulent linage in clinical *S. aureus* infections.

Virulence and antibiotics resistance are two notable aspects to evaluate isolates inducing clinical infections. Antibiotics resistance has been deemed as a hotspot in research of clinical infections for many decades. Generally, isolates with high virulence might be susceptible to the most common antibiotics, while multi-drug resistant isolates exhibit lower virulence, which is named “fitness cost” in related research [54–56]. However, highly virulent heterologous strains have come under scrutiny in recent years, and even the concept of transformation from antibiotics to anti-virulence therapies has been posited [17,57]. Our data show that methicillin susceptible isolates exhibited lower resistance to the first-line antibiotics, while more virulence genes presented in the MSSA, and they exhibited higher hemolysis capacity and higher adhesion ability for epithelial cells, indicating that MSSA exhibits higher virulence in comparison to MRSA. Additionally, MSSA isolates were more likely to cause skin and soft tissue infections, which was more common in community associated *S. aureus* infections, thus more attention should be paid to the MSSA isolates in the prevention and control of infections.

As the dominating clonotype of *S. aureus* infections in Shanghai, China, ST5 exhibited independent evolutionary dynamics in MRSA isolates and MSSA insolates, moreover, ST5 MSSA as an emerging subtype exhibited hypervirulence in comparison to MRSA isolates in this project. However, this research has some limitations. The isolates we collected in this phylogenetic analysis were limited to one territory hospital in Shanghai. Evolutionary reconstruction of isolates in multiple centres might provide more information for our understanding of ST5 *S. aureus*. Additionally, transcriptome or proteomics data of these *S. aureus* isolates could provide more proof of virulence or antibiotics resistance than our NGS data genetically. Further, additional studies for other dominating or emerging clonotype isolates will be required to be characterized for fully reconstructing the evolutionary dynamics of *S. aureus* infections in Shanghai, China.

## Supplementary Material

Figure_S2.jpg

Figure_S1.jpg

Supplementary_Table_1-revised.xlsx
