# Supplementary figures and images for: Increasing prevalence of hypervirulent ST5 methicillin susceptible Staphylococcus aureus subtype poses a serious clinical threat
Source: Emerg Microbes Infect. 2021 Jan 17;10(1):109–22. doi: 10.1080/22221751.2020.1868950 (PMC7832517; doi:10.1080/22221751.2020.1868950)

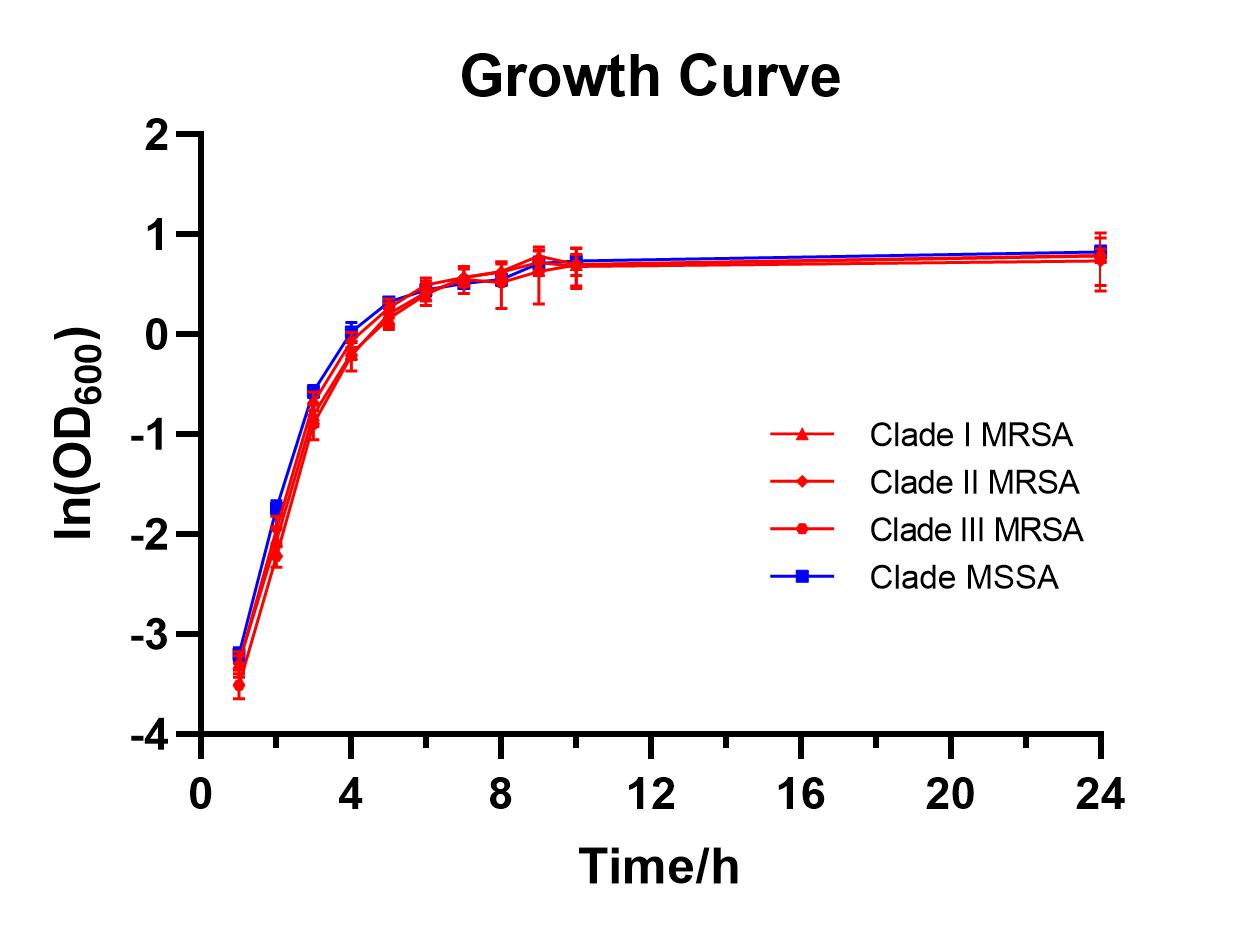

Supplement: Figure_S2.jpg [file TEMI_A_1868950_SM7370.jpg]

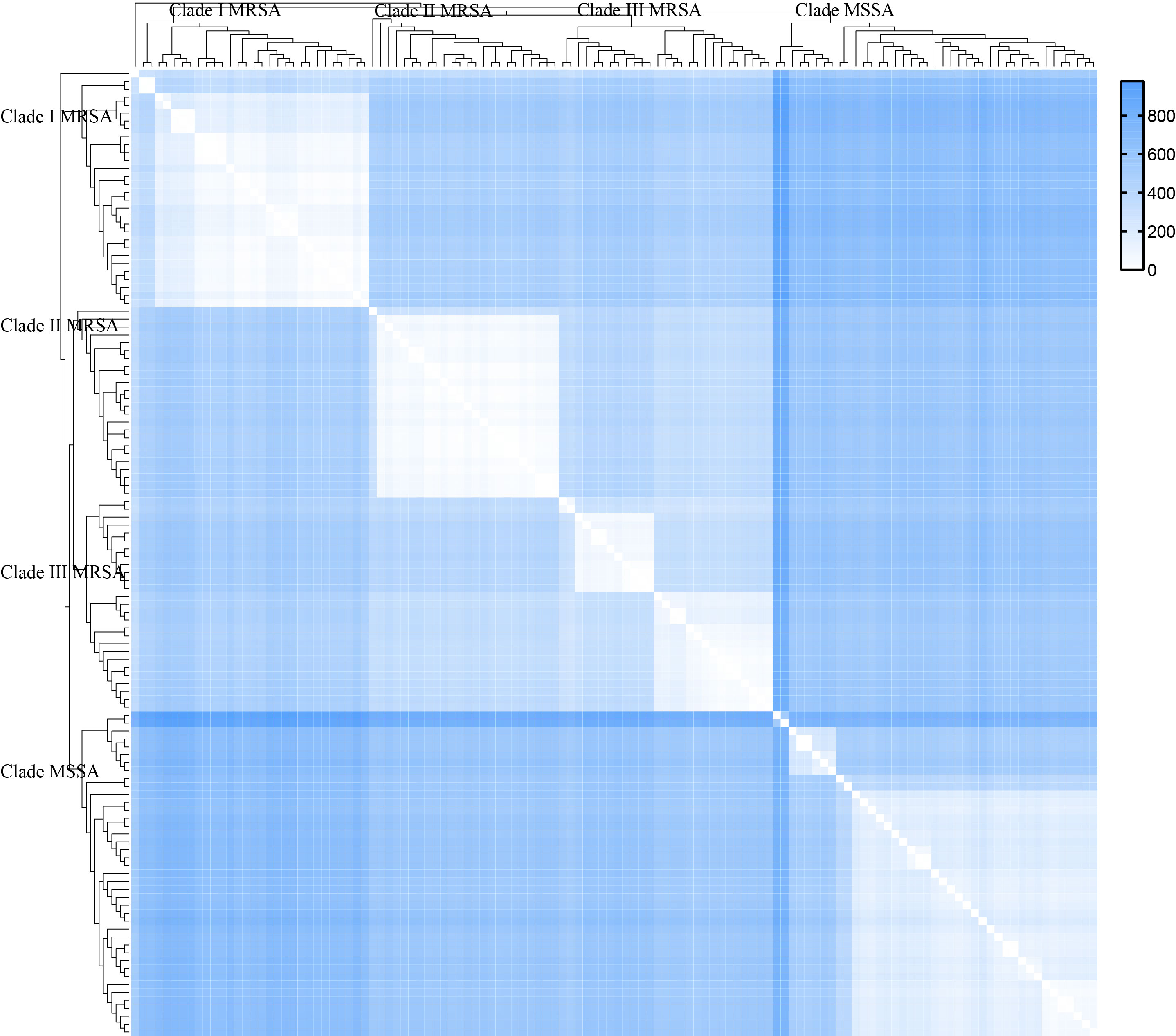

Supplement: Figure_S1.jpg [file TEMI_A_1868950_SM7369.jpg]
